# A Fiber-Tip Label-Free Biological Sensing Platform: A Practical Approach toward *In-Vivo* Sensing

**DOI:** 10.3390/s150101168

**Published:** 2015-01-09

**Authors:** Alexandre François, Tess Reynolds, Tanya M. Monro

**Affiliations:** 1 The Institute for Photonics and Advanced Sensing (IPAS) and ARC Centre of Excellence for Nanoscale Biophotonics (CNBP), the University of Adelaide, Adelaide SA 5005, Australia; E-Mails: tess.reynolds@adelaide.edu.au (T.R.); tanya.monro@adelaide.edu.au (T.M.M.); 2 University of South Australia, Adelaide SA 5000, Australia

**Keywords:** fiber optics sensors, biological sensors, microcavities, laser resonators

## Abstract

The platform presented here was devised to address the unmet need for real time label-free *in vivo* sensing by bringing together a refractive index transduction mechanism based on Whispering Gallery Modes (WGM) in dye doped microspheres and Microstructured Optical Fibers. In addition to providing remote excitation and collection of the WGM signal, the fiber provides significant practical advantages such as an easy manipulation of the microresonator and the use of this sensor in a dip sensing architecture, alleviating the need for a complex microfluidic interface. Here, we present the first demonstration of the use of this approach for biological sensing and evaluate its limitation in a sensing configuration deprived of liquid flow which is most likely to occur in an *in vivo* setting. We also demonstrate the ability of this sensing platform to be operated above its lasing threshold, enabling enhanced device performance.

## 1. Introduction

Over the last decade, whispering gallery modes (WGMs) have found applications in label-free optical biosensing, enabling operation down to the single molecule level [1,2] and also miniature laser sources [3–5], waveguides [6], filters [7] and mechanical [8,9] and temperature [10,11] sensors. Generally, WGMs can be described as light being trapped within a resonator by total internal reflection, circulating along the inner surface and returning in phase after a single or multiple round trips to satisfy the resonance conditions [12]. Multiple resonator geometries have been reported in the literature, ranging from rings/toroids [13] and spheres [14] to cylinders and capillaries [15,16]. The spectral position of the resonances is dictated not only by the resonator geometry (diameter, sphericity) and optical properties but also by the refractive index of the environment surrounding the resonator [12], making this phenomenon particularly interesting for label-free biosensing applications.

A key parameter of such resonators is the quality factor (Q) which effectively describes how many round trips a photon can undergo within the resonator before being lost by absorption or scattering. WGMs can exhibit extremely low losses; when the refractive index contrast at the resonator boundaries is high, the radius of curvature of the resonator exceeds several wavelengths and the intrinsic scattering and surface roughness is small [15], the Q factor can reach several millions, as demonstrated for silica spheres and toroids [13,17]. While such high Q factor resonators can exhibit outstanding sensing performance, their utilization remains restricted in practice. For high Q factor resonators, light must typically be coupled into the resonator through the evanescent field of carefully aligned fiber taper [12,14] or a prism [18]. These approaches are limited by the requirement to maintain a precise gap between the resonator and the tapered fiber or prism to stabilize the position of the resonance [19], realistically rendering these platforms unfit for non-laboratory applications such as point of care or *in vivo* diagnostics.

**Figure 1.**
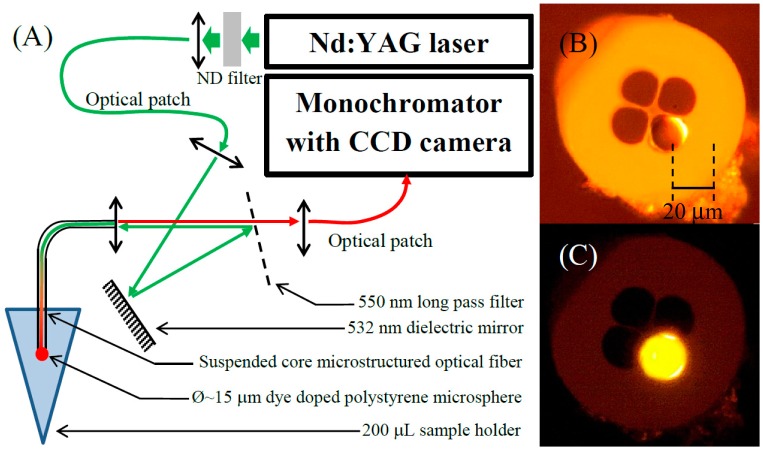
(**A**) schematic of the optical setup; (**B**,**C**) bright field and fluorescence images of a 20 µm diameter dye doped polystyrene microsphere positioned onto the tip of a suspended core silica fiber respectively (Ø_core_ = 4 µm, Ø_hole_ = 17 µm).

**Figure 2.**
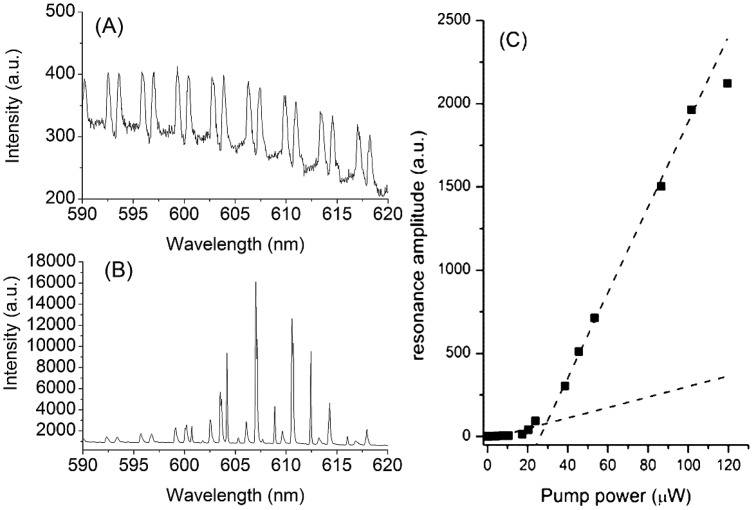
(**A**,**B**) WGM spectra below and above the lasing threshold respectively; (**C**) resonance amplitude as function of the pump power.

Our approach is to combine WGMs as transduction mechanism using an active spherical resonator integrated onto the tip a suspended core Microstructured Optical Fiber (MOF) to create a label-free biosensing platform with the potential application for *in vivo* biosensing. A single dye doped microsphere is located onto one of the holes at the tip of a suspended core fiber as shown in Figure 1B,C. This simple approach takes advantage of the Purcell effect to amplify the emission of light from a gain medium at the resonance frequencies when located within the resonator [20]. Positioning the fluorescent microsphere onto the suspended core microstructured optical fiber tip as seen in the Figure 1A provides a pathway for both the remote excitation and collection of the WGM modulated fluorescence emission seen in the Figure 2A,B, alleviating the need for a cumbersome positioning/coupling scheme [21] as described above. While the use of a high refractive index polymer such as polystyrene (polystyrene = 1.59) as the sphere material enables us to use smaller resonators with higher refractive index sensitivity compared to larger silica spheres, this choice impact the resonator's Q factor, since polymer microspheres have relatively low Q factors [22]. It is important to note that since the analysis of the WGM signal is performed using a standard monochromator to resolve both the spectral position and linewidth of the resonance features, the resolution is ultimately limited by the detection system, which is typically around 4 pm using a 2400 L/mm grating.

*In-vivo* biological sensing is an emerging field with much promise for revolutionary medical diagnostic applications and fundamental breakthrough in biology by enabling measurements to be performed where it has not been possible so far [23]. In this context, optical fiber probes are particularly suited for minimally invasive procedure where the tip of the fiber is rendered active toward the detection of a specific biochemical compound [24,25]. While it is now possible to detect a wide range of biomolecules, ranging from metabolite ions and chemicals [23] such as glucose [25] to enzymes [23,24], the specific detection and quantification of proteins remains an unmet challenge.

Here we evaluate the performance of our fiber tip WGM sensing platform [16,21] for biosensing applications using biotinylated microsphere to specifically capture neutravidin as a first demonstration of the biosensing capabilities of this platform. To mimic the conditions that such a sensor is likely to encounter in an *in vivo* sensing situation, we have deliberately chosen to perform the detection in static conditions and evaluate the detection limit as function of the surrounding neutravidin concentration to assess its suitability for such application. We also investigate how to improve the detection limit by inducing lasing of the WGM to increase the sensor resolution and eventually detection limit.

## 2. Experimental Section

### 2.1. Chemicals

Polystyrene (PS) microspheres (Ø∼20 µm, nPS = 1.59) were purchased from Polysciences, Inc., (Warrington, PA, USA). Nile red fluorescent dye, xylene, poly(allylamine hydrochloride) (PAH), MW ∼15,000 Da and poly(sodium 4 styrenesulfonate) (PSS), MW ∼70,000 Da were received from Sigma-Aldrich (Sydney, Australia), glycerol, >99%, was obtained from Chem-Supply (Gillman, Australia), all chemicals were used as received. N-hydroxysuccinimide (NHS), 1-Ethyl-3-[3-dimethylaminopropyl], carbodiimide hydrochloride (EDC), and ethanolamine hydrochloride (EA), 1 M, were obtained from VWR International (Murarrie, Australia) as a part of the Biacore amine coupling kit. Phosphate buffered saline (PBS) was received in the form of tablets from Sigma-Aldrich (Sydney, Australia) and dissolved in deionized (DI) water yielding a pH of 7.4. Biotin D and neutravidin were received from Sigma Aldrich (Sydney, Australia) and diluted to the relevant concentration in PBS.

### 2.2. Microspheres Preparation

Polystyrene microspheres with a nominal diameter of 20 µm (ΔØ = 0.8 µm, *n* = 1.59) were doped with a fluorescent laser dye (Nile Red, λ_abs_ = 532 nm, λ_em_ = 590 nm) using a liquid two phase system [16,21]. Among the different techniques reported in the literature to either introduce a gain medium within a polymer microsphere [26] or simply coat its surface with either quantum dots or organic dye molecules [27,28], this approach enables high dye content to be loaded within the polymer sphere which is critical for reaching the lasing threshold of the WGMs. The fluorescent dye was first dissolved into xylene until the solubility limit was reached. The resulting solution was poured on top of an aqueous solution of diluted microspheres and left on a magnetic stirrer plate until the xylene had completely evaporated. As xylene and water are immiscible and the fluorescent laser dye used hydrophobic, when the xylene evaporates, the fluorescent dye is transferred into the microspheres that come into contact with the dye solution. After the doping procedure, the microsphere solution was heated above the boiling temperature of the xylene for 1 h to remove any trace of solvent from the microspheres. The microspheres were then washed by centrifugation, the supernatant removed and the lost volume replaced by Millipore water.

### 2.3. Surface Functionalization

Immobilization of relevant proteins onto the microspheres surface was done through the use of polyelectrolyte (PE). Positively and negatively charged PE solutions, polyallylamine hydrochloride (PAH) and polystyrene sulfonate (PSS) respectively were prepared by dissolving 2 mg/mL of either PAH or PSS into a 1 M NaCl buffer. The deposition of the polyelectrolyte onto the microspheres was performed using the layer by layer technique described elsewhere [28,29]. Five layers (PAH/PSS/PAH/PSS/PAH) were deposited onto the spheres. Covalent binding of biotin-D onto the primary amine of the PAH coated sphere was performed in solution using 1-ethyl-3-(3-dimethylaminopropyl) carbodiimide (EDC) and N-hydroxysuccinimide (NHS) as coupling reagents. A 1 mg/mL biotin-D solution in PBS buffer (200 µL) was mixed with both a 1M EDC solution (100 µL) and 1 M NHS (100 µL) and then left incubating with the dye doped sphere solution (∼2.5% volume, 100 µL) for two h. After the immobilization of the biotin-D onto the sphere surface, the microspheres were washed by centrifugation, the supernatant removed and the lost volume replaced by PBS. The passivation of the non-specific binding sites was achieved by incubating the functionalized spheres in a 2.5% casein solution for 24 h. After the passivation step, the sphere were again washed by centrifugation and redispersed in PBS buffer before being stored at 4 °C.

### 2.4. Optical Setup

The optical setup used to operate the sensor is depicted in the Figure 1A. A doubled frequency YAG laser (λ = 532 nm, ∼9 ns pulse duration, 10 Hz repetition rate) was used for the excitation of the active microspheres. The beam emerging from the laser was first spatially filtered using a single mode fiber (Ø_core_ = 8 µm) before being coupled into a silica suspended core Microstructured Optical Fiber (MOF; Ø_core_ = 4 µm, Ø_hole_ = 17 µm). A λ = 550 nm long pass filter was used at almost normal incidence, behaving as a dichroic mirror. This simple optical setup allowed the WGM modulated emission originated from the dye doped resonator, to be recaptured by the MOF, and launch it into a monochromator (600, 1200 and 2400 L/mm grating) equipped with a cooled CCD (2048 pixels) to record the WGM signal, while the sensor head, hence resonator onto the fiber tip, was dipped into a 200 µL sample holder.

### 2.5. Microsphere Attachment onto the MOF Tip

An inverted microscope equipped with a second three axis translation stage was used to position the microsphere onto the MOF tip. A drop of the microsphere solution was deposited onto a glass cover slip and inspected using the inverted microscope while the freshly cleaved MOF end was attached to the second translation stage with the fiber's tip pointing toward the drop of microsphere solution. A microsphere was selected from the many within the drop by qualitatively analyzing its emission spectrum via the confocal excitation and collection provided by the inverted microscope. Once located, the microsphere was brought into contact with the tip of a 80 cm long MOF which was aligned using the independent microscope stage. The microsphere and the MOF tip are both hydrophobic. Thus, once they come into contact, the microsphere tends to adhere to one of the holes of the MOF as shown in both Figure 1B,C. Once the microsphere is attached onto the fiber tip, it remains in this position, allowing easy manipulation of the sensor.

## 3. Results and Discussion

### 3.1. Characterization of the Lasing Behavior of the Dye Doped Resonator

To determine the lasing threshold of the processed dye doped polystyrene microspheres, the excitation power was systematically varied from 5 µW to 100 µW and the corresponding WGM spectra were recorded. The excitation power was calculated assuming a 50% coupling efficiency between the single mode fiber delivering the 532 nm double YAG and the suspended core fiber. This coupling efficiency was measured under the same conditions with a 532 nm CW laser following the same optical pathway. Then, the mode exhibiting the highest lasing intensity was selected and fitted with a Gaussian function. Figure 2A,B shows typical WGM spectra below and above the lasing threshold from the same microsphere while Figure 2C displays the resulting dependence of the peak intensity on the excitation power. On Figure 2A,B the periodic repetition of the first order mode with different mode number and polarization can be seen as previously reported in the literature [4,8,21,22,26].

These figures show that only the modes located around 620 nm are lasing despite the fact the maximum emission of the dye used to dope the resonator is near 590 nm. This phenomenon can be explained by the higher absorption losses of the polystyrene at lower wavelengths [30], which reduces the gain at shorter wavelengths. The evolution of the peak intensity shows clearly two linear regimes with lower slope below and higher slope above threshold, respectively. Therefore, the lasing threshold could be determined by linear fitting of the two linear regions of spontaneous and stimulated emission as indicated by the dash lines in the Figure 2C and subsequent calculation of their intersection. This approach yields a lasing threshold for this resonator of 28 µW, in excellent agreement with previous reported values for a toroidal micro laser [31] and polystyrene microspheres [4,32], both with comparable Q factor. While the non lasing spheres typically exhibit a Q factor, (Q = λ_resonance_/Δλ_resonance_), around 3 × 10^3^ ± 0.8 × 10^3^, the lasing modes are significantly narrower as it can be seen in the Figure 2B, yielding a Q factor above 1.5 × 10^4^ ± 0.5 × 10^4^. As the Q factor is defined as the ratio of the stored energy into the resonator to the energy lost per cycle [33], a higher gain into the resonator, especially upon lasing will increase the stored energy while the lost energy per cycle which is an inherent property of the resonator remains constant, resulting in an increased Q factor. This increase of the Q factor is highly beneficial for sensing purposes as it increase the resolution of the sensor, enabling the detection of smaller changes in the resonance wavelength position [34].

### 3.2. Analysis of the Sensing Performance of the Dye Doped Resonator

To characterize the sensing performance of spheres in a situation that mimics an *in vivo* setting, we have deliberately chosen to simply dip the sensor head, meaning the microresonator attached to the fiber tip, into small Eppendorf tubes without providing any agitation of the solution under study. We do this to reflect the liquid flow that is most likely to occur while performing a measurement *in vivo* instead of using microfluidic flow cells. We sought also to identify the number of PE layers required to achieve a complete surface coverage of the sphere by monitoring the successive deposition of PE layers in real time for non-functionalized spheres. Once a suitable lasing resonator was identified and positioned onto the fiber tip, the sensor head was dipped into a 200 µL Eppendorf tube filled with Millipore water. A reference spectra of the sphere above its lasing threshold was acquired with the highest resolution grating available, yielding a resolution of 4 pm. The sensor head was then removed from the Millipore water and dipped into a second Eppendorf tube filled with PAH. Again another spectra was acquired under the same experimental conditions. After leaving the sensor head immersed in PAH solution for 30 min, it was removed and dipped into Millipore water for rinsing for 10 min before acquiring another spectra. As the concentration of the PE solutions used are very high (2 mg/mL), the coating efficiency/kinetic is thought not to be diffusion limited. This procedure was repeated for the deposition of each PE layer, up to five layers in total, with three different sensors. From each WGM spectra, the spectral position of the resonance features were identify by fitting the resonance peaks with a Gaussian function.

**Figure 3.**
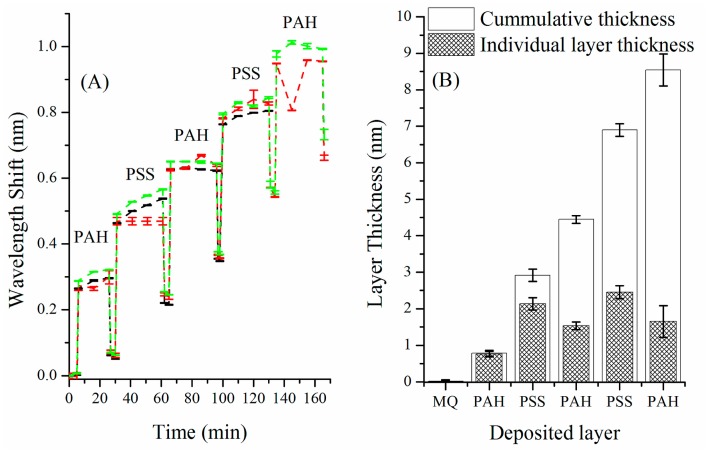
(**A**) Wavelength shift of the WGM of a Ø = 20 µm polystyrene dye doped microsphere for increasing number of deposited polyelectrolyte layers; (**B**) Cumulative and individual layer thickness calculated after the deposition of each polyelectrolyte layer.

The wavelength shift for three different spheres as function of the number of PE layers deposited is shown in Figure 3A. The error bars on Figure 3A are given as the fitting error of the resonance peaks. Figure 3B shows the results of the calculation of the deposited thickness achieved after each PE layer while the error bars on the determination of the layer thickness have been calculated as the standard deviation between the three different equivalent experiments and represent the accuracy in terms of reproducibility rather than the resolution of the sensor itself.

From the wavelength shift, the increase of radius of the sphere can be calculated using Equation (1), where Δλ is the wavelength shift, λ the initial resonance position, ΔR the effective increase in radius, *R* the initial resonator radius, *e* the thickness of the deposited layer, *n_L_* and *n_s_* the refractive index of the deposited layer, typically 1.5 for both PAH and PSS in solution [35], and the resonator respectively:

(1) $$\frac{\Delta \lambda }{\lambda }=\frac{\Delta R}{R}=\frac{n_{L}e}{n_{S}R}$$

The initial radius can be calculated from the peak spacing of two successive modes with the same polarization using Equations (2) and (3):

(2) $$R=\frac{\lambda_{m+1}\times m}{2\pi n_{S}}$$

(3) $$m=\frac{\lambda_{m+1}}{\lambda_{m}-\lambda_{m+1}}+1$$

where *m* is the mode number, λ_m+1_ and λ_m_ the wavelength of two successive first order modes with the same polarization.

It becomes clear from the Figure 3B that the first layer (PAH) is significantly thinner than subsequent PAH layers. This indicates that the first layer doesn't fully cover the resonator which is not surprising and has been previously reported in the literature [28,29]. Nevertheless, the thickness of the first bi-layer (PAH/PSS) is about 3 nm, the second about 6.8 nm, which is good agreement with reported thicknesses of PAH/PSS bi layer deposited under the same conditions [36]. Therefore, we assumed for the subsequent surface functionalization steps that five PE layers would be sufficient to ensure good surface coverage and consequently maximize the density of free amine available for subsequent immobilization of biomolecules.

### 3.3. Demonstration of the Detection of a Specific Interaction

A similar experimental procedure was used to measure the specific binding kinetics of neutravidin onto the biotinylated surface of the resonator and determine what would be the detection limit in an *in vivo* sensing scenario. We choose to use biotin/neutravidin as a specific interaction model because it has been well characterized with other sensing platforms and therefore provides a useful benchmark test. Also it forms the basis of a surface functionalization process we recently used to produce an antibody coating with a specific orientation to increase the corresponding antigen capture efficiency [37].

From the estimate of the increase of radius from the previous section, one can calculate the quantity of adsorbed molecules as follows. The mass per unit surface of the adsorbed molecule is a convenient parameter that does not depend on the geometry of the sensor considered and therefore enables a direct comparison between different techniques and different sensor geometries. The mass per unit surface, *d*, can be calculated using the following equations [2]:

(4) $$d=\frac{M}{N_{A}\sigma_{p}^{-1}}$$

(5) $$\sigma_{p}^{-1}=\frac{n_{S}}{n_{L}}\frac{\alpha_{ex}}{\varepsilon_{0}\left(n_{S}^{2}-n_{m}^{2}\right)}\frac{1}{\delta R}$$

here, *M* is the molecular weight, *N_A_* is the Avogadro number, σ_p_^−1^ the projected area of the adsorbed molecule, α_ex_ its excess polarizability, and n_m_ the refractive index of the medium surrounding the microsphere (*n_m_* = 1.33 for PBS buffer). In a first approximation, the polarizability, α, of the adsorbed molecule which can be calculated by means of the Clausius-Mossotti equation was used instead of the excess polarizability:

(6) $$\alpha =\frac{\varepsilon_{r}-1}{\varepsilon_{r}+2}\frac{3M\varepsilon_{0}}{N_{A}\rho_{m}}$$

here, ε_r_ is the dielectric function of the considered molecule (ε_r_ = *n*^2^ where *n* = 1.5 for most proteins [38], *N_A_* is the Avogadro number and ρ_m_ the mass density (ρ_m_ = 1.37 g·cm^−3^ for most proteins [38]).

#### 3.3.1. Binding Kinetics below the Lasing Threshold

**Figure 4.**
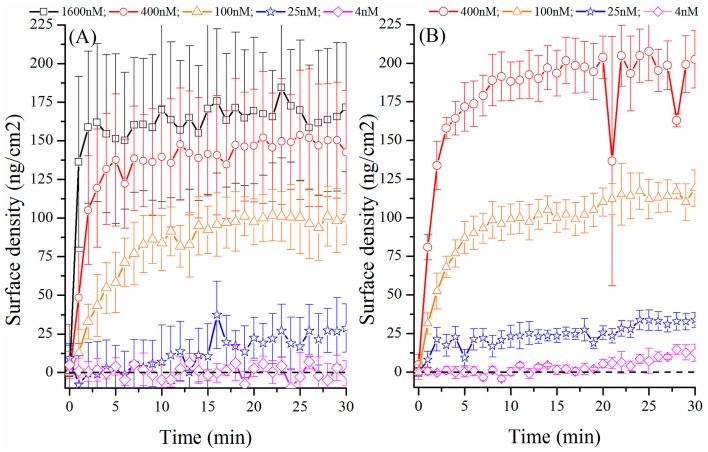
Binding kinetic for neutravidin on a Ø = 20 µm biotin functionalized sphere with sphere operated (**A**) below and (**B**) above the lasing threshold.

As a first test, we replace the pulsed YAG laser pump source with a CW 532 nm solid state laser (2 mW pump power) and triggered the excitation with the acquisition (0.1 s) of the WGM signal performed once per minute to reduce the photobleaching of the organic dye. The objective behind this first set of measurements was to benchmark the binding kinetics when the sensor is operated below its lasing threshold. Figure 4A shows the binding kinetics for the neutravidin (M = 55,000 kDa) with concentrations ranging from 1600 nM (88 µg/mL) down to 4 nM (0.22 µg/mL). As observed in the Figure 4A, the highest concentration (1600 nM) can be easily detected and a saturation of the radius increase occurs within the first minute. We found that the surface density achieved at the steady state with the 1600 nM concentration is about 177 ± 45 ng/cm^2^. This value, within the error, is about half of the density of a full neutravidin monolayer as reported in literature with surface plasmon sensors (445 ng/cm^2^) [37].

An explanation can be found from the observation that approximately one half of the resonator protrudes from the hole on the suspended fiber tip. Given that low concentrations are used during this experiment and that the sample is not subjected to agitation, we infer that the neutravidin did not penetrate into the hole of suspended core fiber, leaving a significant portion of the resonator surface unexposed to the analyte. For the 400 nM concentration, it takes about 5 min to reach the saturation level with a saturation value about half that obtained with the 1600 nM concentration while for the 100 nM concentration, equilibrium is reached after 15 min with an even lower saturation level. Reducing further the concentration a similar trend is found, although the equilibrium regime is then barely reached after 30 min. For the lowest concentration used, 4 nM, no noticeable change of the resonance position was observed beyond the noise level, indicating that this concentration is beyond the detection limit of the sensor. The error bars have been calculated as the standard deviation from the three sets of experimental data acquired for each concentration.

#### 3.3.2. Binding Kinetic above the Lasing Threshold

We repeated the same binding kinetics measurements using the frequency doubled YAG laser as a pump source to operate the spheres above their lasing threshold in an attempt to increase the Q factor and thus improve the detection limit of the sensor defined as the ration between the sensor's resolution and resolution (DL = R/S) [34].

The binding kinetics presented in Figure 4B, obtained with lasing resonators, follow a similar trend as the binding kinetics obtained while exciting the resonators below their lasing threshold. Saturation of the sensor surface upon exposure to the 400 nM neutravidin solution is observed after few minutes while the other concentrations never yielded saturation within the 30 min time frame. However a slight wavelength shift of the WGM, and therefore binding of the neutravidin onto the sensor surface can be observed with the 4 nM concentration, which is not the case with the spheres operated below their lasing threshold. To confirm the specificity of the interaction, the results observed with the lowest neutravidin concentration were repeated using the microspheres without the biotin coating but passivated against non-specific binding with casein following the protocol previously described. In this case, no binding was observed confirming that casein can efficiently block non-specific binding from neutravidin and that for the lowest concentration, the observed wavelength shift is only due to the specific binding of neutravidin onto the biotinylated spheres. This is not surprising considering an effective increase of the Q factor induced by the lasing behavior and the resulting increase of limit of detection. In fact, it can be clearly seen by comparing Figure 4A,B that the error on the measurement which has been determined as the deviation from the mean value from the three sets of independent measurements performed is much lower when the microsphere is excited above its lasing threshold which allows to discriminate the increase of wavelength shift or surface density as presented in both Figure 4A,B from the noise. Despite the absence of flow, which certainly limits the sensor's performance, a limit of detection of 4 nM neutravidin solution is achieved. In term of surface density of bound protein, this is equivalent to 1.3 × 10^15^ molecules/m^−2^ or 120 pg/mm^2^ in an environment deprived of liquid flow. This is approximately 50 times lower than the fiber based WGM sensor reported by Boleininger *et al.* (LOD = 7 × 10^16^ molecules/m^−2^) [39] or half of the detection limit claimed by Chao *et al.* (LOD = 250 pg/mm^−2^) [40].

## 4. Conclusions

In this paper, we have demonstrated the ability of a single dye-doped polymer microsphere to be turned into a microlaser at the tip of a suspended core optical fiber which in turns enables enhanced sensor resolution in individual measurements and can also to be used to detect a specific analyte, neutravidin in this case, down to a concentration of 4 nM (0.20 µg/mL) in an experimental setting deprived of liquid flow, mimicking the conditions anticipated for *in vivo* sensing applications. This result raises the question of the suitability of such sensing platform for the detection of proteins using an antibody/antigen assay, especially when the molecule to be detected is in small concentration. Therefore, at its present stage of development this technology is best suited to the detection of protein that are present in relatively high concentrations such as for example ApoE or clusterin which are stress marker indicators with normal regulation range of 20 to 50 µg/mL [37,41]. To expand the application range of this platform a smaller resonator could offer improved refractive index sensitivity. In this paper, we have used Ø ∼ 20 µm polystyrene microspheres which typically have a refractive index sensitivity of 25 nm/RIU. Reducing the diameter of the resonator to 10 µm should results in a two-fold increase of the refractive index sensitivity [21] although lasing with such microsphere has not been demonstrated yet. A more efficient approach would be to use coupled microspheres to take advantage of the Vernier effect to increase the sensitivity such as proposed by Boriskina [42] or recently demonstrated by Ren *et al.* [43] in coupled capillaries with refractive index sensitivity above 2510 nm/RIU. Beyond the current limitation of this platform, we demonstrate that this simple and robust sensing architecture can be used as a dip sensor, and we envision that it can be used to perform immunoassays in areas that that are present difficult to access with existing sensors.
